# Aucubin, An Active Ingredient in *Aucuba japonica*, Prevents *N*-methyl-*N*-nitrosourea-induced Retinal Degeneration in Mice

**DOI:** 10.3390/molecules24244437

**Published:** 2019-12-04

**Authors:** Eunsoo Jung, Su-Bin Park, Woo Kwon Jung, Hyung Rae Kim, Junghyun Kim

**Affiliations:** 1 Laboratory of Toxicology, Research Institute for Veterinary Science and College of Veterinary Medicine, Seoul National University, Seoul 08826, Korea; ozz79@snu.ac.kr; 2 Department of Oral Pathology, School of Dentistry, Jeonbuk National University, Jeonju 54896, Korea; tnqls309@gmail.com (S.-B.P.); wkjungjbnu@gmail.com (W.K.J.); rlagudfo31@gmail.com (H.R.K.)

**Keywords:** *Aucuba japonica*, aucubin, oxidative stress, retinal degeneration

## Abstract

In the present study, we examined the potent retinoprotective effects of an ethanol-based extract of *Aucuba japonica* (AJE) and its active ingredient, aucubin, on *N*-methyl-*N*-nitrosourea (MNU)-induced retinal degeneration in mice. Retinal degeneration was induced by an intraperitoneal injection of MNU (60 mg/kg). AJE (250 mg/kg) and aucubin (15 mg/kg) were orally administered for 1 week after the MNU injection. Electroretinography (ERG) and histological examinations were performed. Retinal apoptosis and oxidative DNA damage were also quantified. The retinoprotective abilities of AJE and aucubin were also assessed in primary cultured retinal cells. Morphologically, MNU induced a remarkable decrease in the outer nuclear layer, which contains photoreceptor cells. However, this layer was well preserved in the AJE- and aucubin-administered mice. The ERG responses significantly decreased in both a- and b-wave amplitudes in the MNU-injected mice. In the AJE and aucubin-treated mice, ERG responses were significantly increased. In addition, a terminal deoxynucleotidyl transferase dUTP nick end-labeling (TUNEL) assay and immunohistochemical staining for 8-hydroxydeoxyguanosine (8-OHdG) revealed that both AJE and aucubin attenuated MNU-induced photoreceptor cell apoptosis and oxidative DNA damage. Furthermore, the in vitro assay also showed that AJE and aucubin have potent anti-oxidative and anti-apoptotic activities in primary cultured retinal cells. These results indicate that AJE and aucubin have potent retinoprotective effects, and that this retinoprotective activity is as a result of the potency of the bioactive compound, aucubin. These pharmacological characteristics suggest the additional application of AJE or aucubin in the treatment of patients with retinal degenerative diseases.

## 1. Introduction

The dysfunction and degeneration of photoreceptors is the major reason for visual loss [1]. Although the detailed pathophysiology underlying this condition has not been thoroughly understood, its etiological factors include chronic inflammation and oxidative stress. The excessive generation of reactive oxygen species (ROS) in the retina elicits the degeneration of photoreceptor cells and retinal pigment epithelial cells, and this is considered a causative factor of retinal degenerative diseases. Mitochondria are an important endogenous source of ROS, while exogenous ROS is generated under various conditions, such as solar radiation and smoking [2]. The retina has the highest oxygen consumption rate [3]. In the retina, 60% of retinal mitochondria are located in photoreceptor cells [4], which may exacerbate photo-oxidative retinal degeneration [5]. Various anti-oxidative agents have been shown to prevent retinal degeneration in experimental animals and patients with photoreceptor degeneration [6,7]. In the Age-Related Eye Disease Study (AREDS), a mixture of various anti-oxidants (vitamins C and E, β-carotene, and zinc), also known as an AREDS formula, reduced the risk of the progression of macular degeneration by 25% [8].

Medicinal herbs are rich sources of potential anti-oxidative agents. *Aucuba japonica* Thunb. is a traditional Korean medicinal herb and has been used to treat several diseases, such as edema and inflammation [9,10,11]. The leaves of *A. japonica* (AJE) contain various phytochemicals such as aucubin, quercetin, and kaempferol [12,13]. Recently, we reported that AJE and its bioactive compound, aucubin, showed potent pharmacological effects on dry eye disease [14]. The retinoprotective abilities of AJE and aucubin have not yet been described in reports. To elucidate this, we examined the retinoprotective activity of an ethanolic extract of AJE and its active ingredient, aucubin, on a mouse model, to determine retinal degeneration induced by *N*-methyl-*N*-nitrosourea (MNU). The retinoprotective abilities of AJE and aucubin were also assessed in primary cultured retinal cells.

## 2. Results

### 2.1. AJE HPLC Analysis

**Figure 1:**
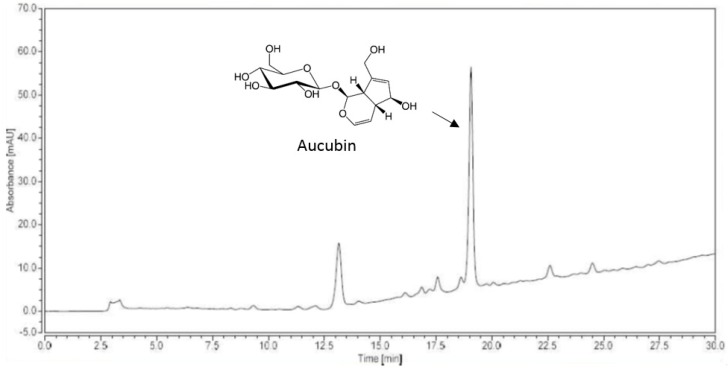
HPLC profile of an extract of *Aucuba japonica*.

The AJE was standardized with high-performance liquid chromatography (HPLC) analysis. The AJE contained 59.7 ± 1.5 mg/g of aucubin (Figure 1). Based on the concentration of aucubin in the AJE, we determined the dose of aucubin for the animal experiment.

### 2.2. AJE and Aucubin Inhibit Histological Retinal Changes

**Figure 2:**
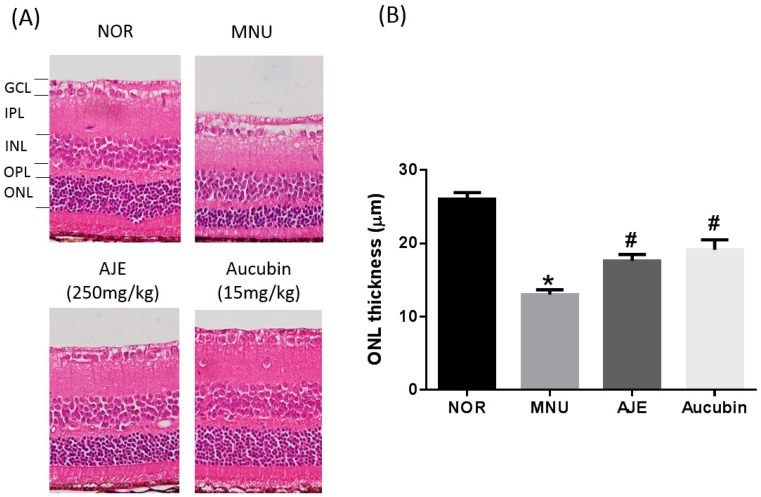
Effects of AJE and aucubin on retinal histological changes. (**A**) Histological changes induced by MNU injection. GCL: ganglion cell layer; IPL: inner plexiform layer; INL: inner nuclear layer; OPL: outer plexiform layer; ONL: outer nuclear layer. (**B**) Quantification of the ONL thickness. Data are expressed as mean ± SEM, *n* = 10, * *p* < 0.01 vs. normal control (NOR) group. # *p* < 0.01 vs. MNU group.

To examine the retinoprotective effects of AJE and aucubin, the thickness reduction of the outer nuclear layer (ONL) caused by MNU-induced photoreceptor cell loss was measured. As shown in Figure 2, all retinal cells were well preserved in the normal control (NOR) groups (ONL thickness: 26.01 ± 3.28 μm). The exposure to MNU induced a significant reduction in the ONL thickness (13.01 ± 3.06, *P* < 0.01). AJE and aucubin stopped photoreceptor cell loss by 40.3% ± 2.5% and 59.8% ± 2.9%, respectively.

### 2.3. AJE and Aucubin Prevent Retinal Dysfunction

**Figure 3:**
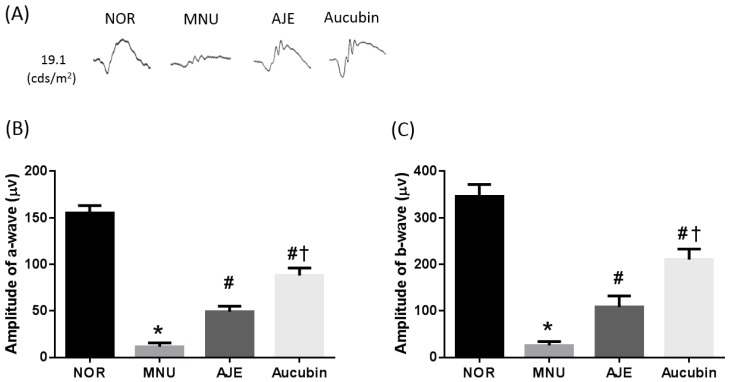
Effects of AJE and aucubin on retinal function. (**A**) Dark-adapted electroretinography (ERG) waveforms. (**B**,**C**) Quantification of the average a- and b-wave amplitudes in scotopic ERG responses. Data are expressed as mean ± SEM, *n* = 10, * *p* < 0.01 vs. NOR group. # *p* < 0.01 vs. MNU group, † *p* < 0.01 vs. AJE group.

To investigate the preventive role of AJE and aucubin on retinal dysfunction induced by MNU exposure, electroretinography (ERG) was applied. The exposure to MNU induced significant reductions of both a- and b-wave amplitudes by 78% and 63%, respectively. However, AJE and aucubin could prevent the decrease of these amplitudes (Figure 3).

### 2.4. AJE and Aucubin Suppress Photoreceptor Cell Apoptosis

**Figure 4:**
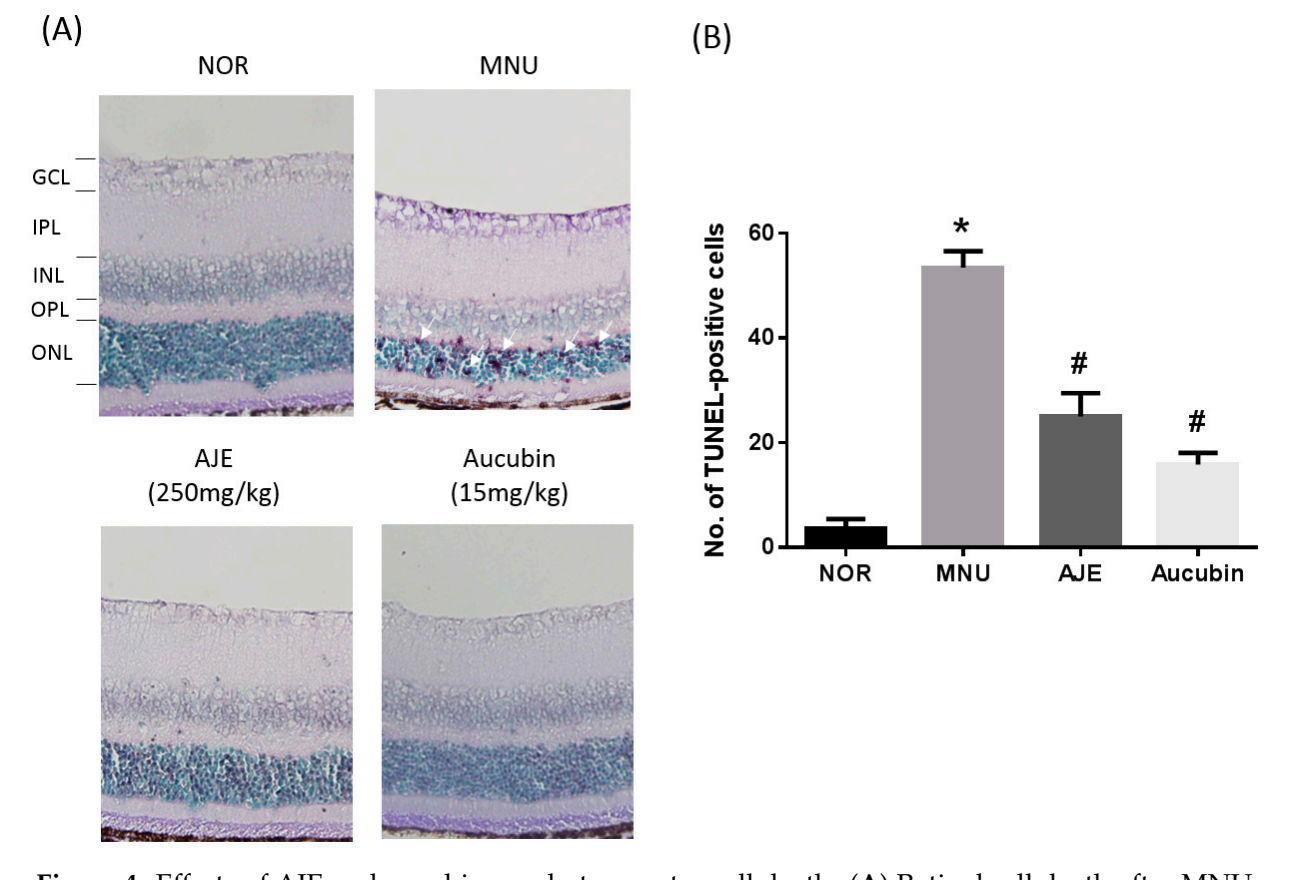
Effects of AJE and aucubin on photoreceptor cell death. (**A**) Retinal cell death after MNU injection was determined by TUNEL staining. The arrows mark TUNEL-positive photoreceptor cells. GCL: ganglion cell layer; IPL: inner plexiform layer; INL: inner nuclear layer; OPL: outer plexiform layer; ONL: outer nuclear layer. (**B**) Quantification of the number of apoptotic cells. Data are expressed as mean ± SEM, *n* = 10, * *p* < 0.01 vs. NOR group. # *p* < 0.01 vs. MNU group.

As shown in Figure 4, no TUNEL-positive cell was observed in any layer of the retina. However, the MNU-injected mice had numerous apoptotic cells, which were primarily detected in the outer nuclear layer. The administration of both AJE and aucubin significantly prevented these apoptotic changes.

### 2.5. AJE and Aucubin Inhibit Oxidative Injury In Photoreceptor Cells

**Figure 5:**
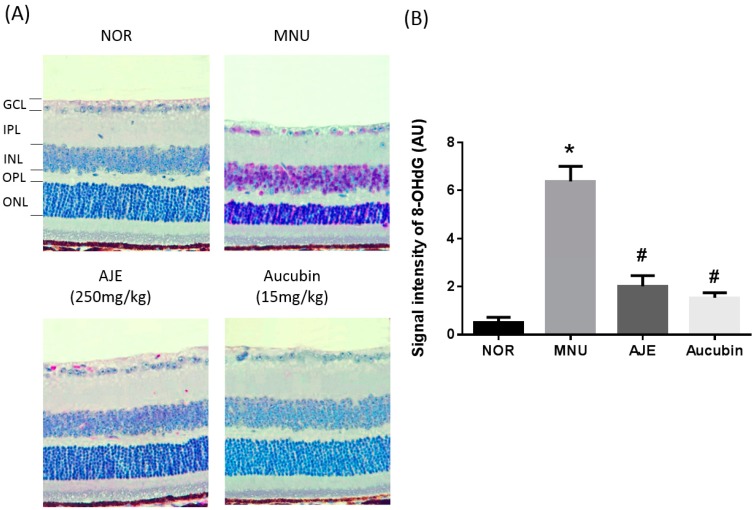
Effects of AJE and aucubin on oxidative DNA damage. (**A**) Immunohistochemical staining for 8-hydroxydeoxyguanosine (8-OHdG), an oxidative DNA damage marker. GCL: ganglion cell layer; IPL: inner plexiform layer; INL: inner nuclear layer; OPL: outer plexiform layer; ONL: outer nuclear layer. (**B**) Quantitative analysis of immunohistochemical staining intensity. Data are expressed as mean ± SEM, *n* = 10, * *p* < 0.01 vs. NOR group. # *p* < 0.01 vs. MNU group.

The formation of 8-hydroxydeoxyguanosine (8-OHdG), induced by the oxidation of guanine, is a well-known marker for oxidative DNA damage [15]. We examined the immunohistochemical staining of 8-OHdG to examine the anti-oxidative role of AJE and aucubin in the retinal tissues. As shown in Figure 5, no immunohistochemical signal for 8-OHdG was detected in the normal mice. However, the nuclei within all the nuclear cell layers were stained intensely with 8-OHdG that may be contributing to oxidative retinal injury. As predicted, 8-OHdG levels were markedly decreased by treatments of AJE and aucubin in these regions, compared to those of the MNU-injected group. Therefore, AJE and aucubin suppress photoreceptor cell apoptosis.

### 2.6. AJE and Aucubin Inhibit Oxidative Injury In Primary Cultured Retinal Cells

**Figure 6:**
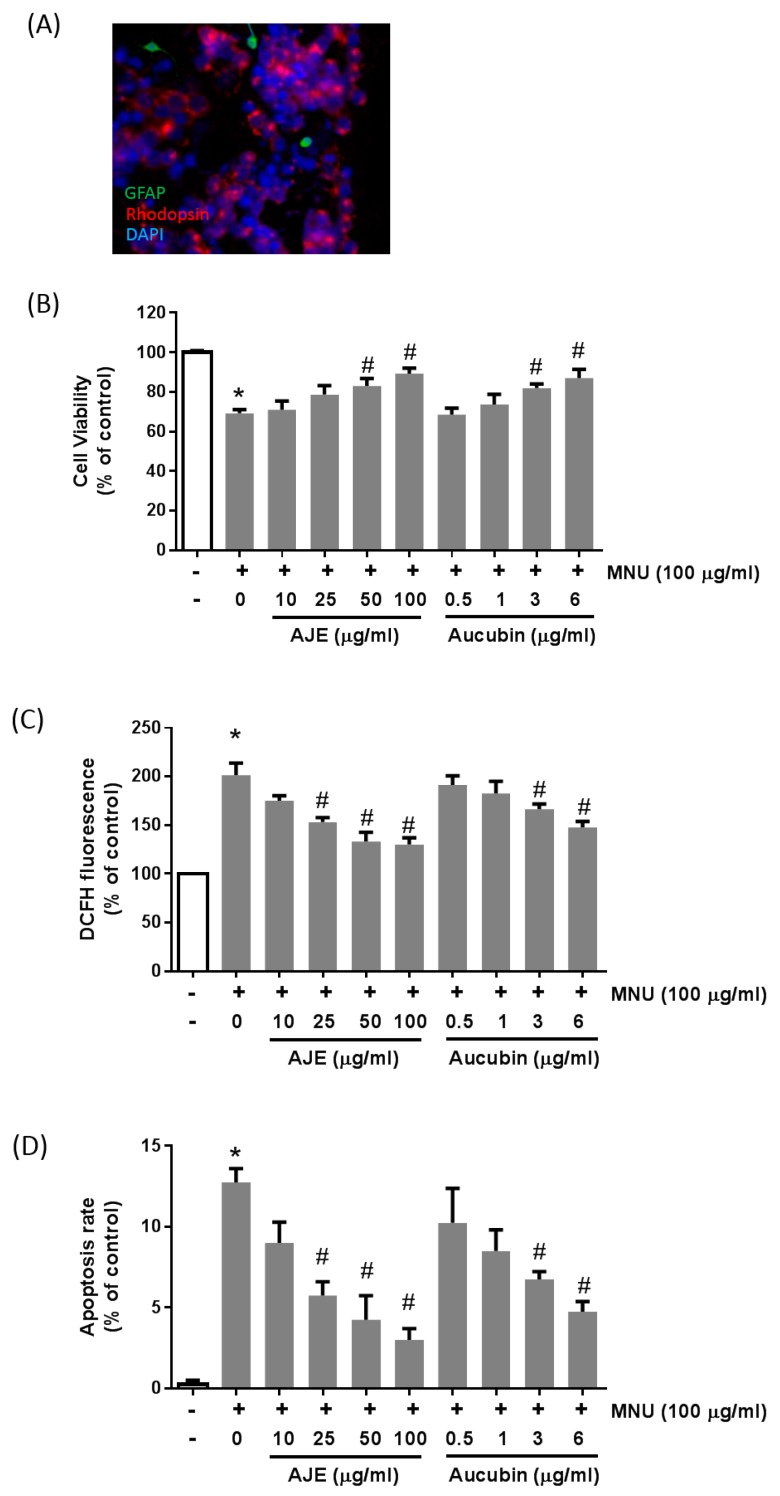
Effects of AJE and aucubin on cell viability, oxidative stress and apoptosis in primary cultured retinal cells. The primary cultured retinal cells were exposed to 100 μg/mL of MNU with AJE or aucubin for 24 h. (**A**) Immunofluorescent staining with anti-glial fibrillary acidic protein (GFAP) and anti-rhodopsin of primary cultured retinal cells. (**B**) Cell viability was measured by the MTT assay. (**C**) Intracellular reactive oxygen species (ROS) generation was detected using dichlorodihydrofluorescein diacetate (DCFH). (**D**) Apoptosis was detected by TUNEL assay. All of the viability is shown as a % of control. Data are expressed as mean ± SEM, *n* = 3, * *p* < 0.05 vs. control cell, # *p* < 0.05 vs. vehicle treated cell.

Primary cultured retinal cells containing photoreceptor cells were exposed to media containing 100 μg/mL of MNU, to confirm the preventive role of AJE and aucubin. MNU treatment elicited cytotoxicity on the retinal cells. The viability of cells incubated with 100 μg/mL of MNU alone was approximately 70% compared to that of the control cells. When the cells were treated with various concentrations of AJE and aucubin for 24 h, the cell viability was recovered in a dose-dependent manner (Figure 6A). In the oxidative stress assay, the dichlorodihydrofluorescein diacetate (DCFH) dye showed dramatically increased levels by MNU, which were significantly decreased by AJE and aucubin (Figure 6B). To confirm whether MNU-induced cytotoxicity was due to apoptosis, the TUNEL assay was performed (Figure 6C). The apoptotic cells were increased by MNU treatment, and restored by AJE and aucubin in a dose-dependent manner.

## 3. Discussion

The mouse model for retinal degeneration induced by MNU has been widely employed to evaluate the effects of novel potent candidates [16,17,18]. In the present study, we aimed to assess whether AJE and its active ingredient, aucubin, have retinoprotective activities. We examined the preventive effects of AJE and aucubin on the functional and histological changes of retinae induced by a single injection of MNU. In the ERG analysis, AJE and aucubin caused increments in both a- and b-wave amplitudes. Similarly, the AJE and aucubin-treated mice showed less histological changes in the ONL layer and weaker expressions of 8-OHdG than those of the MNU-injected mice. Furthermore, the in vitro assay also showed that AJE and aucubin have potent anti-oxidative and anti-apoptotic activities in primary cultured retinal cells. Cumulatively, our findings indicate that AJE could prevent retinal photoreceptors from structural and functional injury induced by a single MNU injection, and that its retinoprotective activity is as a result of the potency of the active ingredient, aucubin.

In this animal model, a reduction in the ONL thickness resulted in degenerative photoreceptor cell loss, which was consistent with a decrease of scotopic ERG responses. Oral administrations of AJE and aucubin inhibited the structural and functional injury of photoreceptor cells. We also demonstrated that AJE and aucubin have a protective effect on the apoptotic death of photoreceptor cells. Apoptosis of photoreceptor cells was characterized in a disease model of retinal degeneration (Jeong et al., 2011; Nakajima et al., 1996; Yuge et al., 1996). These retinoprotective effects of AJE and aucubin can be attributed to their anti-oxidative activities. In the present study, extensive retinal 8-OHdG formation was demonstrated in the MNU-injected mice. AJE and aucubin could suppress retinal 8-OHdG formation. The formation of 8-OHdG induced by the oxidation of guanine is a well-known marker for oxidative DNA damage, and represents an oxidative stress biomarker that is sensitive and stable. A linear correlation between ROS generation and 8-OHdG formation has been reported [19]. Excessive ROS generation in the retinae of MNU-injected mice might lead to the formation of 8-OHdG. Moreover, apoptosis is initiated by DNA damage [20]. This indicates that MNU-induced oxidative DNA damage is responsible for the apoptotic damage of photoreceptor cells. 

Aucubin is a major compound of AJE. Many studies have demonstrated the effects of aucubin against diabetes-induced pancreas injury [21] and carbon tetrachloride-induced hepatic damage [22] through its anti-oxidant activity in several animal experiments. According to these previous reports, it is hypothesized that the pharmacological effects of AJE may be due to the anti-oxidative and retinoprotective effects of aucubin. 

This is the first study to provide evidence that AJE and aucubin have retinoprotective effects in vivo. In addition, AJE and aucubin suppressed oxidative DNA damage and apoptosis in photoreceptor cells in the MNU-induced retinal degeneration model. Further studies may be required to determine the feasibility of using AJE or aucubin for the treatment of patients with photoreceptor degeneration.

## 4. Materials and Methods

### 4.1. AJE Preparation

The leaves and stems of *A. japonica* were cultivated and collected in Geoje, Kyungsangnamdo, South Korea. Chonbuk National University’s (Jeonju, South Korea) herbarium has the voucher specimen (No. JBNU-AJE2018). The leaves (700 g) and stems (350 g) of *A. japonica* were extracted with 30% ethanol (10.5 L) at 85 °C. The extraction took 3 h with a 175 g sample gotten by concentration and freeze-drying. The AJE was qualitatively and quantitatively assessed using high-performance liquid chromatography (HPLC).

### 4.2. Animals and Experimental Disign

To induce retinal degeneration, male C57BL/6 mice (6 weeks old, Orient Bio, Seoul, Korea) were intraperitoneally injected with MNU (60 mg/kg; Sigma, St. Louis, MO, USA). After the injection of MNU, the mice were randomly divided into three groups of 10 mice, as follows: firstly, the MNU-injected mice (MNU); secondly, the MNU-injected mice treated with AJE (250 mg/kg body weight); thirdly, the MNU-injected mice treated with aucubin (15 mg/kg body weight). AJE and aucubin in distilled water were administered via oral gavage for 1 week. NOR mice were given the same volume of vehicle. All procedures were approved by the Institutional Animal Care and Use Committee (Approval No. 19-015).

### 4.3. Electororetinoragphy

After the 12 h dark adaptation, the electroretinography (ERG) was recorded under dim red light. The mice were anesthetized with isoflurane, and their pupils were dilated with 1% tropicamide. A contact lens electrode was placed on the cornea, with a reference subdermal electrode in the scalp. A ground subdermal electrode was placed in the tail. Scotopic ERG signals were obtained using a RetiPort ERG System (Roland Consult, Brandenburg, Germany) with a photic stimulator (10-msec duration 19.1 cd·sec/m^2^ stimulus, Grass Technologies, Warwick, RI, USA).

### 4.4. Histopathology

At necropsy, with the eyes fixed and embedded in 4% paraformaldehyde and paraffin, respectively, retina sections 4 μm thick were made. The sections were then deparaffinized, rehydrated, and stained with hematoxylin and eosin (H&E). The ImageJ program (National Institutes of Health, Bethesda, MD, USA) was applied in calculating the thickness of the ONL.

### 4.5. TUNEL and Immunohistochemical Stainings

Retina sections 4 μm thick were made. The sections were then deparaffinized, rehydrated, and treated. For the TUNEL staining, the sections were stained with an In Situ Cell Death Detection kit (Roche Biochemicals, Mannheim, Germany) according to the manufacturer’s protocols. For the immunohistochemical staining, the sections were incubated with an anti-8-OHdG antibody (Santa Cruz Biotechnology, California, CA, USA) for 1 h at 37 °C. An Envision kit (DAKO, Carpinteria, CA, USA) detected the signal, which was visualized with aminoethylcarbazole. The number of TUNEL-positive cells was counted, and the immunohistochemical signal intensity was calculated using an ImageJ program (National Institutes of Health, Bethesda, MD, USA).

### 4.6. Primary Retinal Culture

The primary retinal cultures were prepared according to a published protocol [23]. Briefly, retinae were dissected from C57BL/6 mice (Orient Bio, Seoul, Korea) and dissociated by papain for 15 min at 37 °C. Neurobasal medium (Gibco, Carlsbad, CA, USA) with ovomucoid (Sigma, St. Louis, MO, USA) and DNase (Sigma, St. Louis, MO, USA) was added, and the cells were centrifuged at 100 × g for 8 min. The cells were resuspended in this medium without DNase. The primary retinal cells were maintained in a neurobasal medium with L-glutamine and a B27 supplement (Gibco, Carlsbad, CA, USA). Primary retinal cells were identified by the immunostaining for rhodopsin (a marker for rod photoreceptor cells) and glial fibrillary acidic protein (GFAP, a marker for Müller glial cells). Our cultured cells contained 65% rod photoreceptor cells and 28% Müller glial cells.

### 4.7. Cell Viability, Apoptosis and Oxidative Stress Assays

The primary retinal cells were plated (1 × 10^4^ cells/well) in triplicate into 96-well plates for 24 h, and mediums were exchanged to different concentrations of AJE and aucubin with or without MNU (100 μg/mL) and incubated for 24 h. Then, cell viability was examined using a cell proliferation assay reagent (MTS assay kit, Promega Corporation, Madison, WI, USA). Oxidative stress was detected using the fluorescent probe, dichlorodihydrofluorescein diacetate (DCFH), as previously described [24]. Apoptotic cells were detected by TUNEL staining.

### 4.8. Statistical Analysis

A one-way analysis of variance and Tukey’s multiple comparison test were applied for group data analysis. A statistically significant difference was indicated by a *p*-value less than 0.05.
